# Primary Care Physician Perceptions of the Impact of CMS E/M Coding Changes and Associations with Changes in EHR Time

**DOI:** 10.1007/s11606-025-09400-1

**Published:** 2025-02-18

**Authors:** Natalya Maisel, Robert Thombley, Christine A. Sinsky, Kathleen Blake, J. Marc Overhage, Kevin Grumbach, Rachel Willard-Grace, Lindsey Carlasare, Julia Adler-Milstein

**Affiliations:** 1 University of California San Francisco, San Francisco, CA USA; 2 American Medical Association, Chicago, IL USA; 3 The Overhage Group, Zionsville, IN USA

## Abstract

**Background:**

In 2021, CMS changed ambulatory evaluation and management (E/M) documentation requirements to reduce physician documentation burden. No prior work has assessed primary care physician (PCP) perceptions of the impact of these changes.

**Objective:**

To determine whether physicians both perceived and objectively experienced a reduction in documentation time.

**Design:**

We used cross-sectional and longitudinal survey data of PCPs and linked it to electronic health record (EHR) data. The setting was a large academic medical center (UCSF Health).

**Participants:**

Eighty-seven PCPs.

**Main Measures:**

Physicians completed pre- and post-surveys about whether the CMS changes affected the amount of time and effort they spent on documentation. We compared survey data to objective audit log measures of documentation time per E/M encounter.

**Key Results:**

PCPs perceived spending less time and effort on documentation of Review of Systems (84% reported a moderate or significant decrease) and History of Present Illness and Physical Exam (41%) after the E/M coding change. Further, we found a shift towards PCPs perceiving that they spend more time on clinically meaningful documentation. We did not find a significant association between perceived and actual changes in documentation time (*b*= −0.01, *s.e.*=0.019, *p*=0.666 for History of Present Illness and Physical Exam; *b*= −0.01, *s.e.*=0.021, *p*=0.780 for Review of Systems). Limitations of the study included using data from a single site and a relatively small sample of PCPs.

**Conclusions:**

PCPs perceived spending less time and effort on documentation after implementation of the 2021 CMS E/M coding changes, changes intended to reduce documentation burden. Using physician perception as an indicator can potentially contribute to understanding the impact of federal policies aimed at reducing documentation burden.

## INTRODUCTION

High levels of physician time spent in the electronic health record (EHR) have been shown to contribute to physician burnout.^1,2^ In response, on January 1, 2021, CMS implemented substantial changes to evaluation and management (E/M) documentation requirements to reduce the physician documentation burden. Major changes included eliminating history of present illness (HPI), physical exam (PE), and review of systems (ROS) as required elements in CPT^®^ code level selection. Large-scale studies using measures of EHR time derived from secondary data have examined whether the revised requirements were associated with reductions in documentation time. Among samples of both national Epic users and Cerner users, there was evidence of only small reductions.^3–5^

However, a key limitation of both studies was that they did not assess *perceived impact* of the changes. It is possible that physicians perceived meaningful reductions in documentation effort—i.e., the cognitive workload associated with complying with the coding requirements—even if the EHR-derived measures of time showed only small reductions. A reduction in required documentation effort would support the intent of the new regulations. It is also possible that reductions in documentation in the targeted domains (HPI/PE, and ROS) freed up time that physicians used for higher-value documentation in other domains. If this is the case, we would expect physicians to report that documentation focuses more on clinically relevant information as opposed to billing requirements—another intent of the regulations.

To directly examine these two possibilities, we undertook a survey of primary care physicians (PCPs) at the University of California, San Francisco (UCSF). Our first research question (RQ1) was whether PCPs perceived changes in the amount of time spent documenting HPI, PE, and ROS due to the E/M coding changes. We also examined Assessment and Plan as an “untreated” comparison because this category was not targeted in the E/M coding changes regulation. Our second research question sought to understand the relationship between perceived impact and EHR-based measures, first by assessing if EHR-based measures of time spent on documentation reflected a reduction after the guideline changes consistent with the national-scale studies (RQ2a), and then whether EHR-based measure changes were consistent with perceived changes (RQ2b). Our third research question (RQ3) was whether the E/M coding changes were associated with perceived changes in the extent to which PCPs felt that they documented primarily for clinical care purposes versus for billing purposes.

## METHODS

### Sample and Setting

Our sample included PCPs at a large, urban academic medical center (UCSF Health). The PCPs worked in the 10 comprehensive primary care practices that vary in size from 5 to 25 physicians: 3 general internal medicine, 1 family medicine, 1 pediatrics, 2 geriatrics, and 3 mixed primary care specialties. This sample had a stable EHR environment with Epic in use since 2012. These physicians respond to an annual survey asking about varied dimensions of their work and practice experiences, which we modified to address our specific research questions. Specifically, we used data from the 2020 (pre) and 2022 (post) surveys. Both surveys were administered using Qualtrics and have been described in prior studies.^6^ The pre-survey was administered in February of 2020 and the post-survey was administered in March of 2022. Based on data availability, the samples for each research question differed slightly. For RQ1, our sample included the 87 PCPs who completed the post survey (72% response rate). For RQ2, our sample included the subset of PCPs (*n*=64) with post-survey data and ambulatory EHR metadata during two periods: (1) pre-E/M coding changes (July 2020–Dec 2020), and (2) post-E/M coding changes (Jan 2021–Dec 2021). For RQ3, our sample included the subset of PCPs (*n*=56) who completed the survey at both timepoints.

UCSF conducted a training session to inform PCPs on changes to E/M coding requirements in December of 2020. While sessions were delivered to all specialties, presentation content remained largely consistent. Physicians received training in the simplified E/M code selection as determined by documentation by time or medical decision making. This included information on categorizing visit complexity as straightforward, low, moderate, or high in three domains: the complexity of problems, the complexity of data, and complications/risk. Based on this matrix of factors, the presentation demonstrated how to select the corresponding E/M code.

The presentation further informed physicians on the elimination of sections such as HPI as key components for code selection. PCPs were presented case studies to enhance understanding, detailed documentation mistakes to avoid, and clarification on how E/M documentation fits into the patient visit workflow. The training additionally provided PCPs with contacts as a resource for ongoing support during this transition. The authors can provide training materials upon request.

### Survey Measures and Analyses for RQ1 and RQ3

Survey items were included in the annual System Transformation Evaluation Project (STEP) survey. We used one item from the existing STEP measures from 2020 and 2022, and we added four new items in 2022 for this study. We collaborated closely with the team responsible for administering the STEP survey, who have previously detailed the survey’s construction.^6^ We worked with the STEP team to develop questions regarding E/M coding changes that were added to the 2022 administration. These new items were piloted by the STEP survey lead with UCSF PCPs. To assess how familiar the physicians were with the coding changes, the 2022 survey asked, “To what extent are you familiar with the CMS changes to E/M documentation requirements that took effect January 1, 2021?” with response options of “1=Not at all,” “2,” “3=Somewhat,” “4,” and “5=To a great extent.” Next, to address RQ1 and assess whether the E/M coding changes affected the perceived amount of time and effort spent on documentation, the 2022 respondents were asked “To what extent have the CMS changes to E&M documentation requirements changed the amount of time and effort you spend on each of the following categories of documentation in the past year?” for targeted documentation domains: (1) History of Present Illness and Physical Examination, and (2) Review of Systems. The 2022 survey also asked about (3) Assessment and Plan in order to examine an “untreated” comparison since this category was not targeted in the E/M changes regulation. Response options included: “1=Significant decrease,” “2,” “3=No impact,” “4,” and “5=Significant increase.” For each of these questions, we created summary statistics describing the distribution of responses using the 5-point scale.

To address RQ3 and assess changes in the purpose of documentation, we utilized the existing STEP item from the 2020 and 2022 surveys which asked, “How would you rate your current efforts on clinical documentation?” (RQ3) using a 10-point scale ranging from “1=Predominantly for billing purposes” to “10=Mostly clinically-relevant information for patient care.” We performed a paired *t*-test comparing the pre- and post-survey responses on the 10-point documentation scale.

Physicians also reported on the survey their gender, years worked at UCSF Health, and percent of time spent on clinical service. These questions were used as demographic controls in our regression model. Table 1 reports summary statistics for survey questions.

**Table 1:** Primary Care Physician Sample Characteristics

PCP sample (*n*=87)	Source	Mean ± SD (Median) or number (%)	
Gender (*n*=84)	Survey		
Male		24 (28.6%)	
Female		58 (69.1%)	
Other		2 (2.4%)	
Years worked at UCSF Health (*n*=86)	Survey		
Less than 1 year		8 (9.1%)	
1–2 years		8 (9.1%)	
3–5 years		17 (19.3%)	
6–10 years		17 (19.3%)	
More than 11 years		36 (41.9%)	
Percent clinical service (vs. research, admin, other) (*n*=85)	Survey		
<25%		27 (31.8%)	
26–50%		20 (23.5%)	
51–75%		22 (25.9%)	
76–100%		16 (18.8%)	
Perceived measures			
Current efforts on clinical documentation (*n*=56)	Survey	2020	2022
1=Predominantly for billing purposes		4 (7.1%)	3 (5.4%)
2		4 (7.1%)	0 (0.0%)
3		3 (5.4%)	4 (7.1%)
4		7 (12.5%)	6 (10.7%)
5		7 (12.5%)	5 (8.9%)
6		9 (16.1%)	6 (10.7%)
7		7 (12.5%)	13 (23.2%)
8		6 (10.7%)	12 (21.4%)
9		6 (10.7%)	4 (7.1%)
10=Mostly clinically relevant for patient care		3 (5.4%)	3 (5.4%)
Extent to which primary care physicians were familiar with the changes to E/M coding requirements (*n*= 87)	Survey		
Not at all (1)		3 (3.5%)	
(2)		6 (6.9%)	
Somewhat (3)		17 (19.5%)	
(4)		33 (37.9%)	
A great deal (5)		28 (32.2%)	
EHR-based measures (*n*=64)	EHR-derived from metadata		
EHR documentation minutes per E/M encounter (across all physician weeks in pre- and post-periods)		30.48 ± 14.27 (31.15)	

### EHR Measures and Analyses for RQ2

To assess EHR-based measures of documentation time and how they relate to perceived measures (RQ2), we first created a measure of time spent on E/M documentation per E/M visit using EHR metadata. EHR documentation time was determined by calculating cumulative total minutes spent writing E/M progress notes during the focal week. This measure sums all time the physician spent editing any note labeled a progress note, for any visits assigned a CPT code in the range of 99201-99205 or 99211-99215, from the moment the first keystroke associated with the note is pressed, until the note is saved, for all editing sessions of the note within the focal week. We used a timeout at 60 s of inactivity when calculating documentation time. We calculated weekly measures of time spent documenting E/M visits in the EHR divided by the number of E/M visits in the week to produce a weekly measure of documentation time (minutes) per E/M visit. In the pre- and post-sample periods, physician-weeks were excluded if there were no E/M visits in the week; additional weeks were excluded if documentation time per visit was an extreme outlier (below the 1^st^ percentile or above the 99^th^ percentile; excluded 2% of weeks). A total of 4502 physician-weeks were included.

To examine change in the EHR-based measures (RQ2a), the median E/M documentation time per E/M visit was calculated for each physician in each time period. A paired *t*-test was conducted to examine change from the pre- to post-period.

To examine whether changes in EHR-based measures were associated with perceived changes (RQ2b), we used EHR-based measures to predict the dependent variable of perceived change in time spent on documentation. The first predictor in the model was a “change score” calculated by subtracting the pre-E/M median documentation time from the post-E/M median documentation time (i.e., how much the EHR documentation time changed from pre to post). In addition to the change score, we added a predictor in the model to focus on the physicians’ “starting level” of documentation time — i.e., whether they were above or below the median in documentation time in the pre-period before the changes were implemented. We controlled for gender, years at UCSF, and time spent on clinical activities.

### Robustness Tests

Because we observed that physicians who were more familiar with the coding changes were more likely to report a decrease in time and effort spent on the documentation of Review of Systems (*r*=−0.28, *p*=0.026; Appendix Table 3), we repeated all analyses limited to those who were familiar with the E/M coding changes (“4” or “5” on the 5-point Likert scale).

### Role of the Funding Source

Funding was provided by the American Medical Association, which employs or employed 3 of the authors (C.S., L.C., and K.B. [retired]). UCSF researchers conducted the study and analyzed the results in collaboration with AMA co-authors.

## RESULTS

### Sample Characteristics

As shown in Table 1, 69% of PCPs identified as female and more than 60% had been working at UCSF Health for more than 5 years. Physicians generally had clinical service as only one part of their FTE (other time was spent on research, admin, etc.): Only 19% reported spending more than 75% time on clinical service (Table 1). Physicians were familiar with the changes to E/M coding requirements, with 90% at least somewhat familiar (“3” or greater on Likert scale) and 70% very familiar (“4” or “5”) (Table 1).

### RQ1: Perceived Impact by Documentation Domain

**Figure 1:**
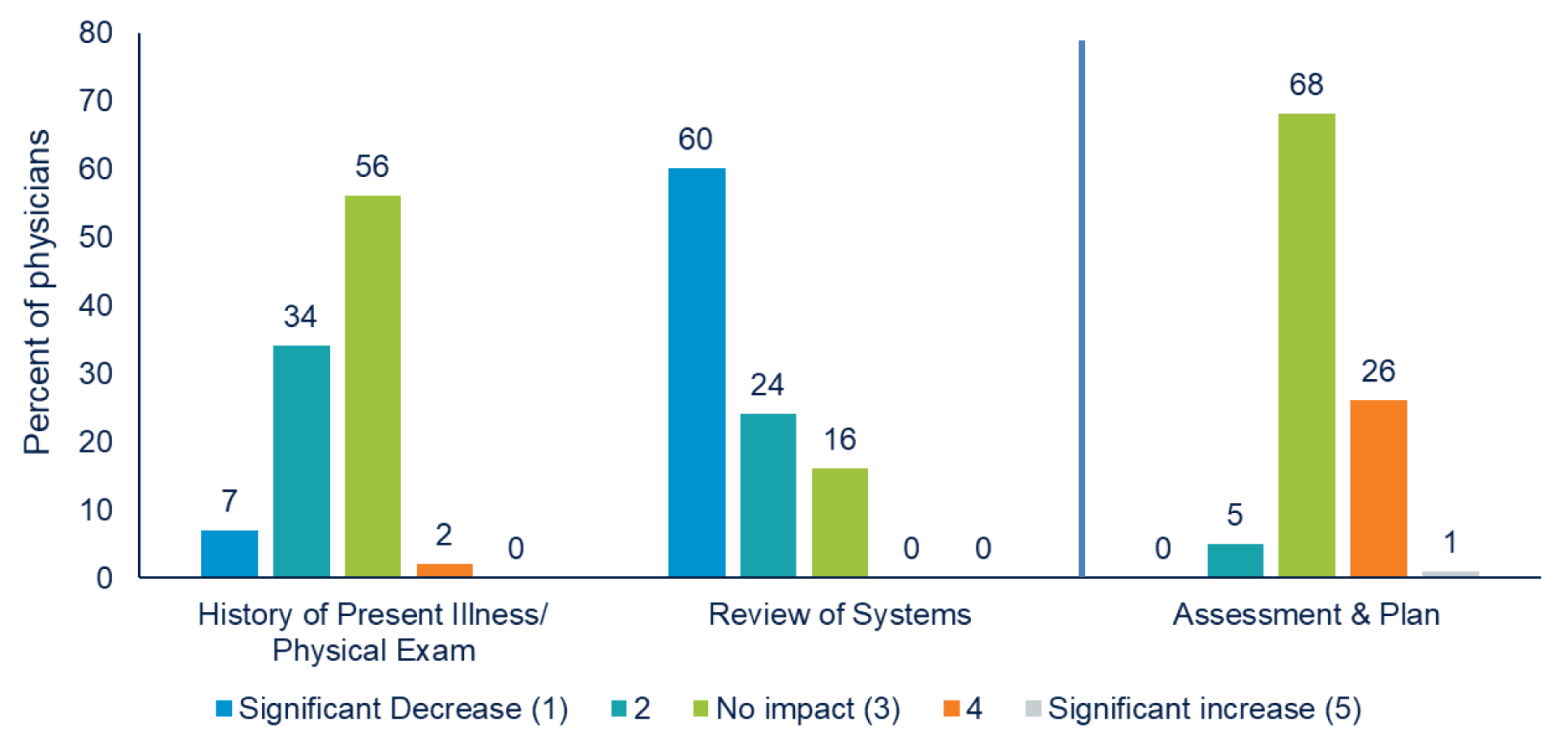
Primary care physicians’ perceived impact of CMS E/M guideline changes on the time and effort spent documenting: History of Present Illness and Physical Exam, Review of Systems, and Assessment and Plan (*n*=85). History of Present Illness and Physical Exam and Review of Systems were targets of the E/M changes, and Assessment and Plan was not.

For HPI/PE documentation, 7% reported a significant decrease in time and effort spent, 34% reported a moderate decrease, 56% reported no change, and 2% reported a moderate or significant increase (Fig. 1). For ROS, 60% of physicians reported a significant decrease, 24% reported a moderate decrease, 16% reported no change, and 0% reported a moderate or significant increase (Fig. 1). For Assessment and Plan, 0% reported a significant decrease, 5% reported a moderate decrease, 68% reported no impact, and the remaining 27% reported a moderate or significant increase (Fig. 1).

### RQ2a: EHR-Based Measures of Reduction in Time Spent on Documentation

On average, median time spent on documentation decreased by 1.16 min (SD = 5.60) from the Pre-E/M changes period to the Post-E/M changes period (31.55 vs 30.39 E/M documentation minutes per E/M encounter), which was a marginally significant decrease in line with the national studies (*t*=1.66, *p*=0.051).

### RQ2b: EHR-Based Measures Associated with Perceived Changes

As seen in Table 2, the EHR-based change score was not a significant predictor of perceived changes in documentation time for either HPI/PE (*b*=−0.01, *p*=0.666) or ROS (*b*=−0.01, *p*=0.780). For HPI/PE, physicians who spent more time (above the median) on documentation in the pre-E/M coding changes period were more likely to report perceived decreases in their time spent on HPI/PE due to the E/M coding changes (approximately a half-point difference on the 5-point scale) (*b*=−0.43, *p*=0.046). This was not statistically significant for ROS (*b*=−0.15, *p*=0.522) (Table 2).

**Table 2:** OLS Regression Models Predicting Perceived Decreased Documentation Time due to E/M Coding Changes

Variable^1^		History of Present Illness and Physical Exam				Review of Systems			
		*coef*	*s.e.*	*t*	*p*	*coef*	*s.e.*	*t*	*p*
Change in pre-E/M to post-E/M average time spent documenting in the EHR		−0.01	0.019	−0.43	0.666	−0.01	0.021	−0.28	0.780
Above the median pre-E/M average time spent documenting in the EHR		−0.43	0.212	−2.05	0.046	−0.15	0.233	−0.64	0.522
Gender	Female	0.31	0.214	1.45	0.153	0.09	0.235	0.37	0.710
Years at UCSF	2 years or less	REF				REF			
	3–5 years	−0.42	0.412	−1.02	0.315	−0.40	0.454	−0.88	0.384
	6–10 years	−0.26	0.442	−0.58	0.564	−0.58	0.486	−0.92	0.240
	More than 11 years	0.28	0.441	−0.63	0.529	−0.45	0.485	−0.92	0.360
Time spent in clinical service	<25%	REF				REF			
	26–50%	0.16	0.245	0.68	0.503	0.02	0.269	0.07	0.944
	Over 50% (combined 51–75% and 76–100% for sample size)	−0.27	0.262	−1.04	0.303	0.14	0.288	0.50	0.618
Constant		3.32	0.617	5.38	<0.001	2.03	0.678	3.00	0.004

^1^Variables “Change in pre-E/M to post-E/M average time spent documenting in the EHR” and “Above the median pre-E/M average time spent documenting in the EHR” were measured from EHR audit log data. “Gender,” “Years at UCSF,” and “Time spent in clinical service” were from the survey instrument

### RQ3: Perceived Changes in Documenting for Clinical Care Purposes

In 2020, physician reports of their current efforts on clinical documentation were leaning towards clinically relevant, with over half of respondents rating a 6 or higher on the 10-point scale (Table 1). When we compared 2020 and 2022, we found that perceptions of current documentation moved further towards clinical relevance: 5.70 in 2020 to 6.30 in 2022 (difference = 0.607, *s.e.* = 0.306, *p*=0.026), a relative increase of 11%.

### Robustness Tests

When limiting the sample to physicians who reported being aware of the E/M coding regulations, a larger proportion of physicians reported decreases in documentation time spent on HPI/PE (48% reporting a significant or moderate decrease compared to 41% of the full sample) and on ROS (90% reporting a significant or moderate decreased compared to 84% of the full sample). Regression analyses predicting perceived decreases in documentation time for those who were aware of the regulations produced similar results compared to the full sample. Changes in EHR time spent on documentation were not related to perceived decreases, and physicians who spent more time (above the median) on documentation in the pre-E/M coding changes period were more likely to report that they decreased their time spent on HPI/PE due to the E/M coding changes (marginally significant; *b*=−0.45, *p*=0.069). Results for ROS remained non-significant.

## DISCUSSION

This study is novel in conducting a survey of PCPs to generate a more complete understanding of the impact of the CMS E/M documentation requirement changes intended to reduce EHR burden. Burden can be considered in terms of both time and cognitive effort. We found that median objective time spent on documentation did not significantly decrease. However, we found that PCPs *perceived* spending less time and effort on documentation of ROS and HPI/PE after the E/M coding change. Perceptions in reduction of effort may reflect a decrease in the cognitive load required of physicians to meet the new simplified coding requirements compared with the previous labyrinthine complexities. Further, we found a shift towards PCPs’ perceptions that they spend more time on clinically meaningful documentation.^4^ Previous work found little change in documentation time after the E/M guideline changes^3–5^; the current study reveals that PCP perceptions of these changes may reflect the broader intention of the policy. Our findings suggest that the CPT E/M coding changes, in an environment that invested in ensuring physician awareness of the changes, are achieving at least a portion of their intended impact.

PCPs who reported decreased perceived time and effort spent on documentation tended to have overall higher levels of EHR documentation time at baseline. This suggests that decreases in documentation time are more salient for PCPs who already spend the most time on documentation. It could be that this group had the greatest opportunity for reductions — that is, they were taking the most time to record a detailed HPI/PE and ROS in their documentation and therefore could move away from this under the changed requirements. Further, these physicians may have been more diligent about thorough documentation, such that they were more likely to repurpose the new time to other documentation tasks, which could explain why we did not observe a large reduction in our direct measures of EHR time. This indicates that changes in E/M coding requirements may have had the greatest impact on the group that was most negatively impacted by previous coding requirements. It also suggests that efforts to educate clinicians on the new requirements may be most effectively focused on those with the highest levels of documentation time — an actionable strategy for many health systems with EHR vendors that make this measure available (e.g., Epic Signal, Cerner LightOn).^7,8^

Our study raises the question of how to define success for policy efforts to reduce physician documentation burden. Prior studies that included only measures of EHR time concluded that the 2021 change in E/M documentation requirements had a small effect that was unlikely to be clinically meaningful.^3,5^ By adding perception data, which could be a proxy for cognitive burden, our study found that physicians did report perceived reductions in time and effort spent on the targeted areas. The lack of impact on perceived time and effort for those areas that were not targeted by the regulatory change supports the validity of those perceptions. Physicians in our study also reported improved ability to devote time to clinically relevant documentation. Our results underscore the importance of including physician perceptions of work effort in future assessments of documentation burden reduction efforts.

Nonetheless, it is also important to reflect on the magnitude of reduction in perceptual measures, and to put these in the context of the value of efforts to educate physicians on the new requirements (which require physician time and organizational cost). UCSF chose to heavily invest in training physicians on the new requirements, and the fact that 41% of physicians reported at least a moderate decrease on time spent on HPI/PE documentation and 84% reported at least a moderate decrease for ROS documentation suggests a large-magnitude effect. These results should encourage other organizations to invest in educating physicians on changes intended to reduce documentation burden. To support this, the AMA has made available a range of educational and training materials.^9^ Yet UCSF did incur non-trivial, one-time costs associated with the education efforts, suggesting the need to identify low-cost strategies to help physicians easily learn and adapt to changing documentation regulations. There is an opportunity for EHR-embedded tools that could offer “on the job” training in line with clinician workflow.

### Limitations

Limitations of our study include that our data come from a single health system — a large academic medical center — and include a relatively small sample of primary care physicians. Physicians generally were parttime in their clinical work. Additional research is needed to explore generalizability to community practice settings and other specialties. It is important to note that the effects of the E/M coding guidelines are intertwined with the training UCSF physicians received during their implementation such that this training could account for some or all of the effects we observed.

Our survey measures could have been subject to phenomena such as social desirability bias, which may have influenced physicians to report spending less time on documentation, and mean reversion, which may have contributed to the observed perceived reductions in documentation time compared to baseline. The 10-point Likert scale item on clinical documentation may have posed interpretation challenges for physicians, as the scale’s endpoints are not truly bipolar. While we acknowledge this limitation, our primary focus was on measuring changes over time in this item rather than interpreting the absolute value on the Likert scale. Additionally, the survey question on burden included language that addressed both “time” and “effort,” such that we cannot separate the two dimensions in our interpretation of the results.

In the EHR data, we could not distinguish time spent on different documentation domains. It is possible that reductions in documentation in the domains affected by the CMS regulations freed up time that physicians used for higher-value documentation in other domains.

## CONCLUSION

Our results suggest that PCPs perceived spending less time and effort on documentation of ROS and HPI/PE after a major federal effort to reduce physician documentation burden. PCPs also perceived that they spend more time on clinically meaningful documentation. Given the modest reductions in EHR-measured time, going forward it will be important to continue to use physician perception as a key source through which to assess federal policies targeting documentation burden reduction.

## Appendix

**Table 3:** Correlations among the survey items (Pearson’s *r*) and objective EHR documentation time (*n*=64)

	Perceived change in time/effort: History of Present Illness/Physical Exam	Perceived change in time/effort: Review of Systems	Perceived change in time/effort: Assessment & Plan	Familiar with CMS changes
Perceived change in time/effort: Review of Systems	**r=0.28*, p=0.029**	--	--	--
Perceived change in time/effort: Assessment & Plan	r=-0.02, p=0.898	r=-0.10, p=0.450	--	--
Familiar with CMS changes	r=-0.20, p=0.123	**r=-0.28*, p=0.026**	r=-0.04, p=0.743	--
Pre-post change in objective EHR E/M documentation per E/M visit	r=-0.04, p=0.758	r=-0.06, p=0.659	r=0.24, p=0.060	r=-0.11, p=0.386

^*^*p*<0.05

## Declarations

### Disclaimer

The opinions expressed in this article are those of the authors and should not be interpreted as AMA policy.
